# Development and psychometric validation of the faithful insight scale among Muslim university students

**DOI:** 10.3389/fpsyg.2026.1913719

**Published:** 2026-10-12

**Authors:** Abdulaziz Faleh Al-Osail, Mashael Nasser Ayed Al-Dosari, Mohamed Sayed Abdellatif, Ali Lamouchi, Bandar Almailabi, Mohamed Ali Nemtallah

**Affiliations:** 1 Department of Curricula and Teaching Methods, College of Education-King Faisal University, Al-Ahsa, Saudi Arabia; 2 Department of Educational Sciences, College of Education in Al-Kharj, Prince Sattam Bin Abdulaziz University, Al-Kharj, Saudi Arabia; 3 Department of Psychology, College of Education in Al-Kharj, Prince Sattam Bin Abdulaziz University, Al-Kharj, Saudi Arabia; 4 Department of English Language and Literature, College of Science and Humanities, Prince Sattam bin Abdulaziz University, Al-Kharj, Saudi Arabia; 5 Department of Education, Islamic University of Madinah, Madinah, Saudi Arabia; 6 Educational Psychology and Statistics Department, Faculty of Education, Al-Azhar University, Dakahlia, Egypt

**Keywords:** factor analysis, faithful insight, Islamic psychology, Muslim university students, psychometric validation, scale development

## Abstract

Despite growing recognition of spirituality’s role in psychological functioning, the literature remains critically devoid of psychometrically sound instruments capable of measuring faith-based cognitive insight within Muslim populations, as existing tools are predominantly anchored in Western secular frameworks inadequate for capturing Islamic epistemological dimensions. The present study aimed to develop and psychometrically validate the faithful insight scale (FIS) among Al-Azhar university students. A sequential multi-phase design was employed across two independent samples: an exploratory sample (*N* = 679) and a confirmatory sample (*N* = 843), alongside a retest subsample (*N* = 140). Content validity was established through expert panel review, exploratory factor analysis examined the underlying structure, and confirmatory factor analysis evaluated competing models. Construct validity and reliability were comprehensively assessed. Results revealed a robust four-factor hierarchical structure encompassing constructive interpretation, surface–depth integration, meaning–value perception, and moral conscious awareness, demonstrating excellent model fit, strong convergent and discriminant validity, excellent internal consistency, and adequate temporal stability. The FIS constitutes a culturally grounded and empirically rigorous instrument that authentically integrates Islamic epistemological principles within contemporary psychometric methodology, offering researchers and practitioners a validated tool for advancing theory and evidence-based practice in Islamic psychology and Muslim mental health contexts.

## 1 Introduction

Faithful Insight is conceptualized as a psychological and spiritual concept integrating cognitive awareness, self-reflection, and wisdom grounded in Islamic principles, representing an individual’s capacity to perceive and interpret lived experience through the lens of faith-based understanding derived from the Qur’an and Sunnah. Within the Islamic tradition, faith is understood as a firm conviction deeply embedded in the heart, guiding thought, behavior, and moral discernment (Unus and Caldwell, 2018). Contemporary scholarship has increasingly recognized the intersection of spirituality and psychological functioning, particularly within faith-centered frameworks (Bagis et al., 2024; Pinto et al., 2024). Spirituality has further been identified as a meaningful component of wisdom, encompassing self-reflection, transcendental awareness, and conscious moral orientation (Jeste et al., 2020; Jeserich et al., 2023). Islamic faith principles emphasize self-mastery, discipline, and submission to Allah as foundational dimensions of psychological and spiritual well-being (Kusnandar and Mirza, 2025).

Al-Azhar University students represent a uniquely compelling population for the development and psychometric validation of the FIS, embodying the convergence of rigorous Islamic scholarship and contemporary academic inquiry. Muslim university students have been widely recognized as an appropriate demographic for validating faith-integrated psychological instruments, given their sustained engagement with religious doctrine alongside exposure to diverse intellectual traditions (Bukhori et al., 2022; Hajra and Saleem, 2021; Jamin and Damni, 2024). Within higher education contexts, Muslim students typically aged 18–30 demonstrate meaningful variation in religiosity, academic background, and spiritual orientation, characteristics that strengthen the psychometric robustness of scale validation procedures (Bukhori et al., 2022; Jamin and Damni, 2024). Such diversity ensures that the resulting instrument captures the full spectrum of faith-based cognitive and reflective experiences rather than reflecting a narrow or homogeneous sample (Hajra and Saleem, 2021).

A substantial body of scholarship has demonstrated that mainstream psychometric instruments measuring insight, self-awareness, and cognitive reflection remain predominantly anchored in Western, secular epistemological frameworks that inadequately capture the spiritual and religious dimensions of cognition central to Muslim psychological experience (Dasti and Sitwat, 2014; Ertit, 2023; Haque et al., 2016). The majority of existing religiosity and spirituality scales applied to Muslim populations were either developed within Judeo-Christian theological traditions or represent direct adaptations of Western tools, rendering their construct validity with Muslim respondents fundamentally limited (Chen et al., 2020; Dasti and Sitwat, 2014; Ertit, 2023). Islamic psychology, rooted in Qur’anic epistemology and classical scholarship, conceptualizes cognition, self-reflection, and awareness through distinctly faith-integrated dimensions that secular frameworks systematically overlook (Haque et al., 2016; Mitha, 2020; Tanhan and Young, 2021). This persistent methodological gap underscores the pressing need for culturally and theologically grounded instruments capable of authentically measuring faith-based insight within Muslim populations (Bukhori et al., 2022; Jeserich et al., 2023). Broader culturally integrated frameworks for mental health in Islamic-Asian societies similarly emphasize that psychological constructs cannot be meaningfully assessed independently of the religious and cultural systems in which they are embedded, reinforcing the rationale for developing the FIS as an indigenously grounded rather than adapted instrument (Naeim and Basharpoor, 2026; Narimani and Naeim, 2025).

Despite significant advances in Arabic psychometric research across a range of psychological constructs, including resilience (Fekih-Romdhane et al., 2024b), emotional intelligence (Mohamed et al., 2026), hope (Fekih-Romdhane et al., 2025), and clinical insight (Fekih-Romdhane et al., 2024a), the current literature remains critically devoid of a culturally adapted, psychometrically sound instrument capable of measuring Islamic-based cognitive insight, constituting a profound lacuna in Arab-Islamic quantitative psychological research. Systematic reviews of Islamic psychological instruments have consistently emphasized the urgent necessity for methodologically rigorous tools that authentically integrate Islamic epistemological principles rather than merely adapting Western constructs (Abu Bakar et al., 2025; Haque et al., 2016). Existing Arabic measures of cognitive insight predominantly address secular clinical populations, particularly schizophrenia, without engaging the faith-grounded dimensions of self-reflection and awareness that are intrinsic to Islamic conceptualizations of the self (Fekih-Romdhane et al., 2024a; Haque et al., 2016). This absence fundamentally constrains theory development and evidence-based practice within Arab-Islamic psychological science (Abu Bakar et al., 2025; Obeid et al., 2024).

Al-Azhar University, as the world’s oldest and most globally recognized institution of Islamic scholarship, constitutes an unparalleled contextual setting for the development and psychometric validation of a faith-based psychological instrument such as the FIS. Empirical scholarship consistently demonstrates that the construct validity and cultural authenticity of Islamic psychological scales are substantially strengthened when validation is conducted within environments deeply saturated with Islamic educational traditions, communal worship, and religious practice (Aziz et al., 2025; Ghorbani et al., 2014; Haque et al., 2016). Islamic seminarians and university students immersed in religiously structured academic environments have been shown to demonstrate richer and more nuanced engagement with faith-based constructs than general student populations (Ghorbani et al., 2014; Jamin and Damni, 2024; Saputra et al., 2025). The student body of Al-Azhar University, shaped by centuries of integrated Islamic intellectual and spiritual formation, thus represents the most culturally congruent and contextually authentic population for rigorously evaluating the reliability and validity of an Islamic-grounded psychological measure (Aldahadha and Dwakat, 2024; Aziz et al., 2025; Nazam et al., 2022). This choice of population was made to maximize the likelihood that participants possessed the sustained religious formation necessary to meaningfully endorse faith-based items during initial validation, consistent with evidence that culture and spirituality are transmitted and expressed distinctively within religiously immersed communities (Hammood et al., 2025).

The conceptual foundation of the FIS integrates three interrelated levels of Islamic psychological analysis into a single developmental pathway, addressing the need for an explicit conceptual synthesis rather than a list of loosely related concepts. At the ontological level, nafs (self), qalb (heart), ʿaql (intellect), and rūḥ (spirit) describe the interconnected inner faculties through which a believer perceives and interprets experience. At the developmental level, tazkiyat al-nafs (purification of the self) describes the process through which these faculties are refined and aligned with revealed guidance. At the practice level, murāqabah (self-observation), muḥāsabah (self-accountability), and tafakkur (reflective contemplation) constitute the concrete spiritual disciplines through which tazkiyat al-nafs is enacted. Sustained engagement with these practices is theorized to cultivate baṣīrah, situating ontological structure, developmental process, and daily practice within a single, empirically testable conceptual pathway (Andrabi, 2025).

Faithful Insight is conceptually anchored in baṣīrah, the outcome of this ontological–developmental–practice pathway, and is operationally defined as the capacity to perceive, interpret, and morally evaluate lived experience through faith-based understanding cultivated via tazkiyat al-nafs. This anchoring distinguishes Faithful Insight from related but conceptually distinct constructs: unlike spirituality and spiritual intelligence, which describe a general transcendent orientation or the adaptive application of spiritual awareness (Naeim et al., 2026), Faithful Insight is specifically anchored in Islamic revelatory sources; unlike religious coping, which concerns strategies for managing stress, it describes a stable interpretive capacity rather than a coping response; unlike meaning-making, which is not inherently faith-specific, it is explicitly grounded in Qur’anic epistemology; and unlike Islamic religiosity, which indexes belief and ritual practice, Faithful Insight captures the cognitive-spiritual lens through which lived experience is interpreted (Andrabi, 2025).

Each hypothesized FIS dimension was informed by a specific facet of this framework: Constructive Interpretation reflects tafakkur-based reframing of adversity through ʿaql; Surface–Depth Integration reflects the qalb’s capacity to perceive hidden meaning beneath apparent circumstance; Meaning–Value Perception reflects muḥāsabah applied to evaluating the significance of experience; and Moral Conscious Awareness reflects murāqabah as continuous self-monitoring against revealed moral standards. This a priori mapping, summarized in the conceptual model in Figure 1, guided item generation and provided the theoretical basis for hypothesizing a four-factor structure loading onto a single higher-order Faithful Insight factor corresponding to baṣīrah, which was tested empirically in the analyses reported in Figure 1.

**Figure 1:**
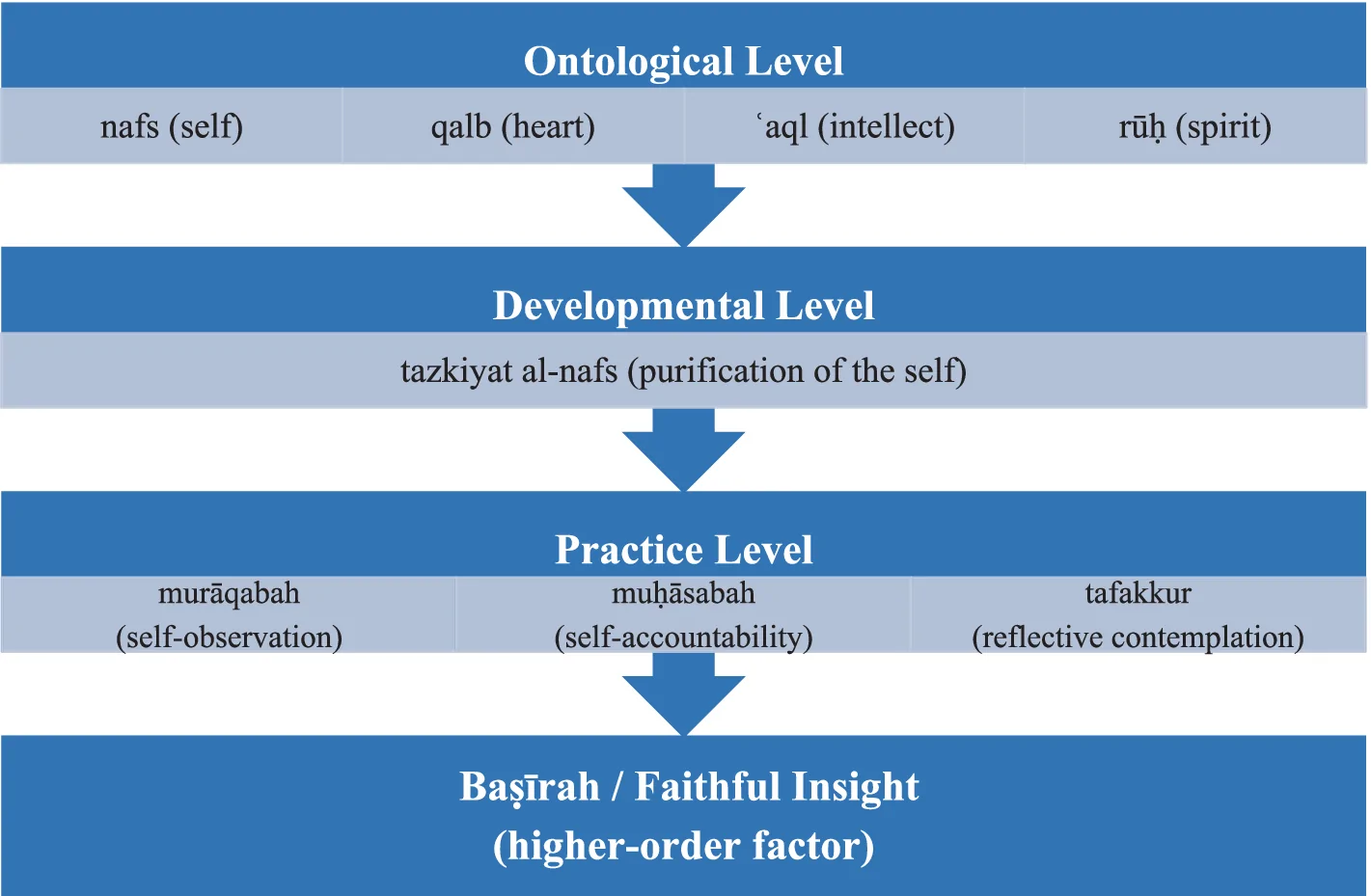
Conceptual model of the ontological–developmental–practice pathway to Baṣīrah (Faithful insight).

The present study aims to develop and psychometrically validate the FIS among Al-Azhar University students through a sequential multi-phase validation design. Specifically, the study seeks to establish the content validity of the scale through systematic expert panel review, to explore and subsequently confirm its underlying factor structure across two independent samples, and to rigorously evaluate its construct validity encompassing both convergent and discriminant dimensions. The study additionally aims to examine the reliability of the FIS through multiple indicators of internal consistency as well as temporal stability, thereby providing comprehensive psychometric evidence for the FIS as a culturally grounded and empirically sound instrument for measuring faith-based cognitive and spiritual insight within Muslim university populations.

## 2 Method

### 2.1 Study design

The present study employed a cross-sectional instrumentation design aimed at developing and psychometrically validating the FIS among Al-Azhar University students. The validation process followed a sequential multi-phase approach encompassing expert content review, Exploratory Factor Analysis (EFA), Confirmatory Factor Analysis (CFA), and reliability estimation. Two independent samples were employed to ensure methodological rigor: the first for scale structure exploration and the second for structural confirmation. A retest subsample was additionally recruited to evaluate temporal stability.

### 2.2 Participants

Two independent samples were recruited from Al-Azhar University students across four faculties during the second academic semester of 2025/2026 (March–April 2026) via Google Forms. The first sample (*N* = 679; mean age = 21.13 years, SD = 1.99) was used for EFA, and the second independent sample (*N* = 843; mean age = 21.06 years, SD = 2.05) was used for CFA. An additional retest subsample (*N* = 140) was drawn 16 days after CFA administration to assess temporal stability. Demographic characteristics of both primary samples are presented in Table 1.

**Table 1:** Demographic characteristics of study participants.

Variable	Category	EFA Sample		CFA Sample	
		Frequency	Percent (%)	Frequency	Percent (%)
Gender	Male	374	55.1%	406	48.1%
	Female	305	44.9%	437	51.8%
Residence	Rural	389	57.3%	469	55.6%
	Urban	290	42.7%	374	44.4%
Major	Scientific	347	51.1%	393	46.6%
	Literary	332	48.9%	450	53.4%
Faculty	Education Boys	200	29.5%	188	22.3%
	Education Girls	152	22.4%	239	28.4%
	Sharia Boys	174	25.6%	218	25.9%
	Humanities Girls	153	22.5%	198	23.5%

*N* = frequency; % = valid percentage of total sample.

Both samples were broadly balanced across gender, residence, and faculty, with only slightly higher CFA-sample representation of female and Education Girls/Humanities Girls respondents, supporting the adequacy of the two samples for the exploratory and confirmatory analyses reported below.

### 2.3 Instrument development

The FIS was constructed to measure faith-based cognitive and spiritual insight among Muslim university students, addressing a recognized gap wherein existing psychometric tools remain anchored in Western secular frameworks inadequate for capturing spiritually grounded dimensions of Muslim psychological experience (Dasti and Sitwat, 2014; Haque et al., 2016), and existing Arabic cognitive insight measures have been developed predominantly for secular clinical populations (Fekih-Romdhane et al., 2024a). The scale’s conceptual foundation integrates classical Islamic constructs — nafs, qalb, ʿaql, and rūḥ — operationalized through the practices of murāqabah, muḥāsabah, and tafakkur within tazkiyat al-nafs (Andrabi, 2025), alongside trait theory and cognitive-behavioral frameworks to enable empirical measurement (Andrabi, 2025). Item generation was guided by this integrated framework and relevant Islamic psychology literature (Bukhori et al., 2022; Ghorbani et al., 2014; Nazam et al., 2022). Items were rated on a 5-point Likert scale from 1 (Strongly Disagree) to 5 (Strongly Agree), with higher scores reflecting greater Faithful Insight. The initial item pool was generated through a combined deductive-literature approach: items were drafted to operationalize each of the four theoretically derived dimensions shown in Figure 1, then refined against terminology and phrasing used in the cited Islamic psychology literature, and written and reviewed in Arabic by the research team, all of whom are native Arabic speakers affiliated with Saudi and Egyptian Islamic higher-education institutions, to ensure linguistic and cultural fit with the target population prior to expert content review.

### 2.4 Content validity

The initial item pool was submitted to a panel of 10 subject-matter experts for content review. Experts were purposively selected to jointly represent the domains most relevant to the FIS: Islamic psychology and Sharia sciences, psychometrics and scale development, and clinical or counseling psychology, and each held a doctoral degree with a minimum of five years of relevant academic or clinical experience at an accredited university. Judges evaluated each item on three criteria: clarity of wording, appropriateness for the target population, and scoring key accuracy. The Content Validity Ratio (CVR) was calculated per item using Lawshe’s formula, with the critical value for a panel of 10 judges set at 0.62. Items failing to meet this threshold were excluded. The overall Content Validity Index (CVI) was subsequently computed as the mean proportion of items rated acceptable across all judges. The final refined item pool retained for psychometric analysis comprised those items meeting both the CVR and CVI standards.

### 2.5 Data analysis strategy

All analyses were conducted using SPSS v.27 and AMOS v.26. Prior to EFA, the KMO measure of sampling adequacy and Bartlett’s test of sphericity were computed to confirm factorability of the correlation matrix. EFA was then performed on the first sample using PAF with Promax oblique rotation, given the theoretical expectation of intercorrelated dimensions. Factor retention decisions were guided by eigenvalues greater than 1, scree plot inspection, and parallel analysis, with a minimum loading threshold of 0.50 applied. CFA was subsequently conducted on the second independent sample to evaluate competing structural models. Model fit was assessed using *χ*^2^/df, RMSEA, CFI, TLI, GFI, and RMR. Construct validity was evaluated through CR, AVE, and MaxR(H), while discriminant validity was examined via the Fornell–Larcker criterion, whereby AVE estimates were compared against squared inter-factor correlations.

CR is an index of internal consistency reliability rather than validity; convergent validity was instead indexed by AVE (recommended threshold ≥ 0.50) and CR (≥ 0.70), discriminant validity by the Fornell–Larcker criterion, and factorial validity by the CFA fit indices reported above, following the thresholds and procedures recommended by Fornell and Larcker (1981) and Hair et al. (2019). Standardized factor loadings were considered salient at ≥ 0.50 (Hair et al., 2019). Internal consistency was estimated using Cronbach’s α, McDonald’s ω, and Guttman’s λ₂. Finally, test–retest reliability was examined via the Intraclass Correlation Coefficient (ICC; two-way mixed-effects model, absolute agreement), the recommended statistic for temporal stability, rather than Pearson correlation, between Time 1 and Time 2 scores in the retest subsample (*N* = 140, interval = 16 days).

### 2.6 Ethical considerations

Participation was entirely voluntary and informed consent was obtained prior to data collection. Anonymity and confidentiality were maintained throughout, with no identifying information linked to individual responses. Data were collected exclusively through Google Forms in accordance with standard ethical guidelines governing psychological research with university student populations.

## 3 Results

### 3.1 Content validity

The initial 24-item pool was evaluated by a panel of 10 expert judges using Lawshe’s CVR, with a critical value of 0.62 for a panel of this size. Item-level CVR values are presented in Table 2. Four items (Items 8, 12, 15, and 23) yielded CVR values below the critical threshold (0.50–0.60) and were therefore removed from the item pool. The remaining 20 items met the CVR criterion and were retained for psychometric analysis. The overall CVI, computed across the criteria of item–sample appropriateness, clarity of wording, and accuracy of the scoring key, was 0.83, indicating an acceptable level of overall content validity for the retained item set.

**Table 2:** Item-level CVR and retention decisions.

Item	CVR	Decision
Item 1	1.00	Retained
Item 2	1.00	Retained
Item 3	1.00	Retained
Item 4	1.00	Retained
Item 5	1.00	Retained
Item 6	1.00	Retained
Item 7	1.00	Retained
Item 8	0.60	*Deleted*
Item 9	1.00	Retained
Item 10	1.00	Retained
Item 11	1.00	Retained
Item 12	0.50	*Deleted*
Item 13	1.00	Retained
Item 14	1.00	Retained
Item 15	0.60	*Deleted*
Item 16	1.00	Retained
Item 17	1.00	Retained
Item 18	1.00	Retained
Item 19	1.00	Retained
Item 20	0.90	Retained
Item 21	0.80	Retained
Item 22	1.00	Retained
Item 23	0.50	*Deleted*
Item 24	1.00	Retained

Critical value for 10 judges = 0.62. Items with CVR below the critical value were deleted.

The overall CVI of 0.83 indicated that the retained 20-item pool met conventional standards for content adequacy, providing a suitable basis for the exploratory factor analysis reported next.

### 3.2 Exploratory factor analysis

The 20-item version of the FIS was subjected to EFA using the first sample (*N* = 679). The Kaiser–Meyer–Olkin (KMO) measure of sampling adequacy was 0.895, and Bartlett’s test of sphericity was significant, *χ*^2^(190) = 5930.35, *p* < 0.001, confirming that the correlation matrix was suitable for factor analysis. Principal axis factoring (PAF) with promax oblique rotation was conducted. Four factors with eigenvalues greater than 1 were extracted, jointly accounting for 55.7% of the total variance prior to rotation. The results of the total variance explained are presented in Table 3, and the parallel analysis results, which corroborated the retention of four factors, are presented in Table 4 and illustrated in Figure 2.

**Table 3:** Total variance explained for the retained factors (promax rotation).

Factor	Eigenvalue	% of variance	Cumulative %	Rotated SumSq loadings
1	7.820	36.9	36.9	3.117
2	1.901	7.4	44.3	2.746
3	1.608	6.0	50.3	2.733
4	1.522	5.4	55.7	2.540

Extraction method, Principal axis factoring. Rotation sums of squared loadings cannot be summed to a total when factors are correlated.

**Table 4:** Parallel analysis results for factor retention.

Factor	Real-data eigenvalue	Mean simulated eigenvalue (95th %ile)	Decision
1	7.820	1.316	Retain
2	1.901	1.258	Retain
3	1.608	1.218	Retain
4	1.522	1.175	Retain
5	0.848	1.145	Do not retain

A factor was retained if its real-data eigenvalue exceeded the 95th percentile of eigenvalues derived from simulated random data of the same dimensionality.

**Figure 2:**
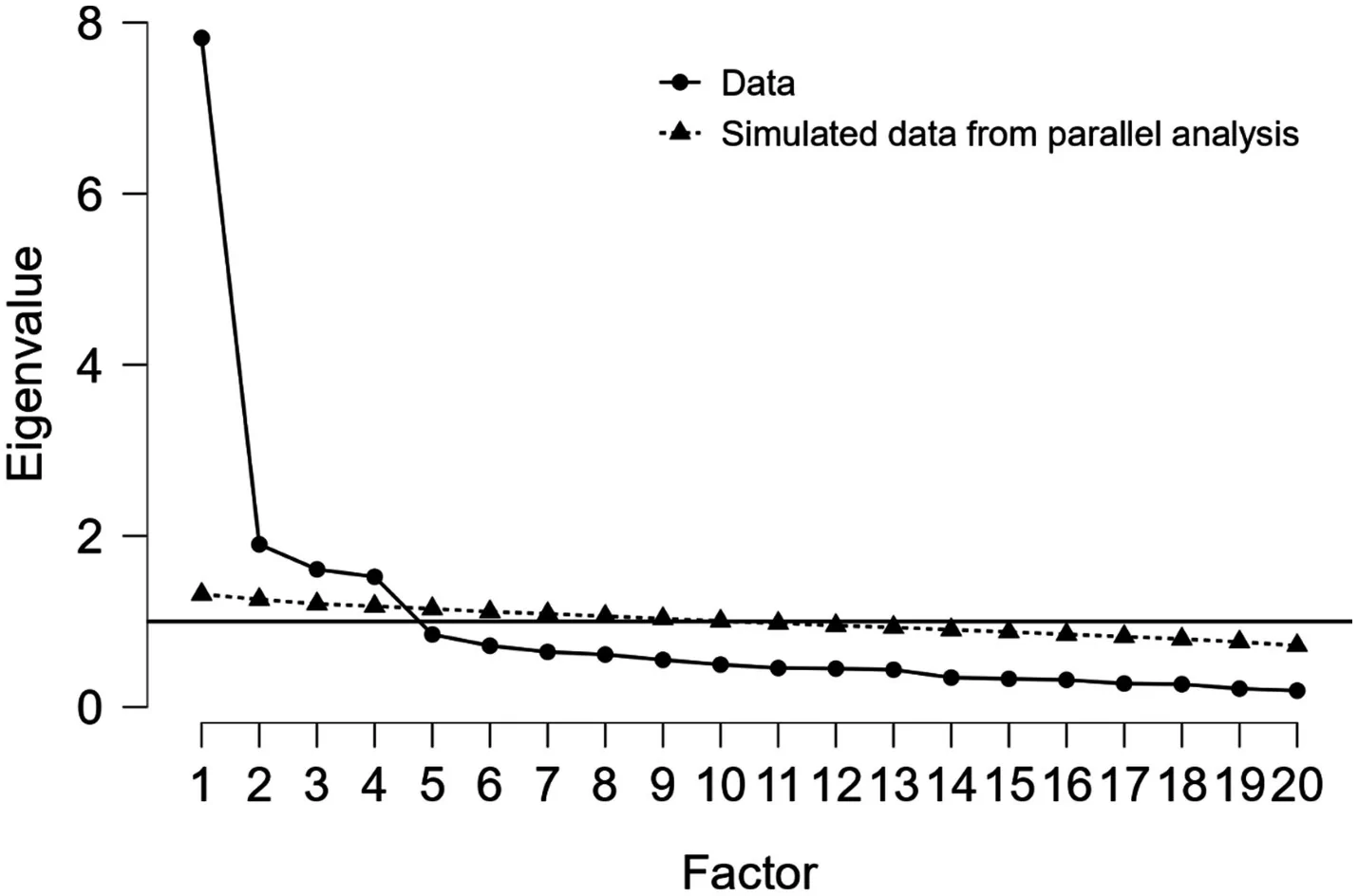
Scree plot with parallel analysis overlay.

Item loadings on the rotated pattern matrix, together with item communalities (h^2^), are presented in Table 5. All 20 items demonstrated a primary loading of 0.50 or greater on their respective factor, with no item exhibiting a salient cross-loading (≥ 0.50) on more than one factor. The four-factor structure aligned with the theoretically proposed dimensions: Constructive Interpretation (6 items), Surface–Depth Integration (5 items), Meaning–Value Perception (5 items), and Moral Conscious Awareness (4 items).

**Table 5:** Pattern matrix: standardized factor loadings and communalities (*h*^2^).

Item	F1	F2	F3	F4	*h* ^2^
Item 16. I view blessings as responsibilities that require gratitude and mindful behavior.	0.791				0.658
Item 11. I see challenges as opportunities for learning rather than mere suffering.	0.731				0.533
Item 14. I believe every experience, even the harsh ones, contains an element that strengthens my character.	0.718				0.511
Item 13. I approach both positive and negative events as parts of my personal development path.	0.568				0.407
Item 12. I try to identify what can help me grow in difficult situations before complaining.	0.565				0.435
Item 15. I strive to transform hardships into future strengths.	0.552				0.467
Item 10. I can integrate practical realities with faith-based guidance without conflict.		0.732			0.558
Item 8. I believe some situations have internal dimensions not visible at first glance.		0.644			0.481
Item 6. I consider long-term outcomes rather than focusing only on the present moment.		0.629			0.488
Item 9. I recognize that wisdom can extend beyond material explanations.		0.622			0.512
Item 7. I balance surface-level causes with deeper meanings when interpreting events.		0.561			0.432
Item 2. I try to understand the value-based dimension of any situation before judging it.			0.799		0.571
Item 3. I believe every event I encounter can contribute to my inner growth in some way.			0.748		0.568
Item 5. I believe that each experience contains a hidden meaning that guides better decisions.			0.702		0.547
Item 4. I view daily situations as opportunities to uncover new insights.			0.616		0.410
Item 1. I feel that events around me carry moral messages beyond their immediate appearance.			0.510		0.405
Item 19. I ensure that my response to situations aligns with the values I believe in.				0.817	0.640
Item 20. I can quickly distinguish between what aligns with my values and what contradicts them.				0.733	0.566
Item 18. I regularly re-examine my intentions when facing morally sensitive choices.				0.695	0.519
Item 17. I notice the ethical dimension of situations before making decisions.				0.643	0.499

Loadings below 0.50 are not displayed and are represented by —. Extraction method, principal axis factoring; rotation method, promax with Kaiser normalization.

The convergence of eigenvalue, scree, and parallel-analysis criteria on four factors, together with a clean simple-structure loading pattern, provided strong preliminary support for the hypothesized four-dimensional model prior to cross-sample confirmation.

### 3.3 Confirmatory factor analysis

CFA was conducted on the second independent sample (*N* = 843) using AMOS v.26 with maximum likelihood estimation. Two competing models were tested: (a) a unidimensional model in which all 20 items loaded on a single latent factor, and (b) a four-factor hierarchical (higher-order) model in which the four first-order factors identified through EFA (Constructive Interpretation, Surface–Depth Integration, Meaning–Value Perception, and Moral Conscious Awareness) loaded onto a single higher-order Faithful Insight factor. The hierarchical model was specified as the theoretically preferred structure *a priori*, consistent with the conceptual model in Figure 1, in which the four dimensions were derived as facets of a single overarching capacity (baṣīrah) rather than as independent constructs; the unidimensional model served as a baseline comparison to establish that this multidimensionality was empirically necessary.

Model fit indices for both competing models are presented in Table 6. The unidimensional model demonstrated poor fit to the data, *χ*^2^/df = 29.01, RMSEA = 0.182, CFI = 0.563, TLI = 0.511, GFI = 0.521, RMR = 0.185. In contrast, the four-factor hierarchical model demonstrated substantially improved and acceptable fit, *χ*^2^/df = 3.98, RMSEA = 0.060, CFI = 0.954, TLI = 0.948, GFI = 0.925, RMR = 0.055. All fit indices for the hierarchical model met or exceeded conventional thresholds for acceptable model fit. The four-factor hierarchical model was therefore retained as the final measurement model for the FIS.

**Table 6:** Model Fit Indices for the Unidimensional and Four-Factor Hierarchical Models.

Fit index	Unidimensional model	Four-factor hierarchical model	Recommended threshold
*χ*^2^/df	29.01	3.98	< 3 (acceptable < 5)
RMSEA	0.182	0.060	≤ 0.06–0.08
CFI	0.563	0.954	≥ 0.90
TLI	0.511	0.948	≥ 0.90
GFI	0.521	0.925	≥ 0.90
RMR	0.185	0.055	Close to 0

CFI, Comparative Fit Index; TLI, Tucker–Lewis Index; GFI, Goodness-of-Fit Index; RMSEA, Root Mean Square Error of Approximation; RMR, Root Mean Square Residual.

Standardized factor loadings for all items in the retained four-factor hierarchical model are presented in Figure 3. All item loadings on their respective first-order factors were statistically significant (*p* < 0.001) and ranged from 0.485 to 0.870. The higher-order loadings of the four first-order factors on the global Faithful Insight factor ranged from 0.673 to 0.745, indicating that each first-order dimension contributed substantially to the overarching construct.

**Figure 3:**
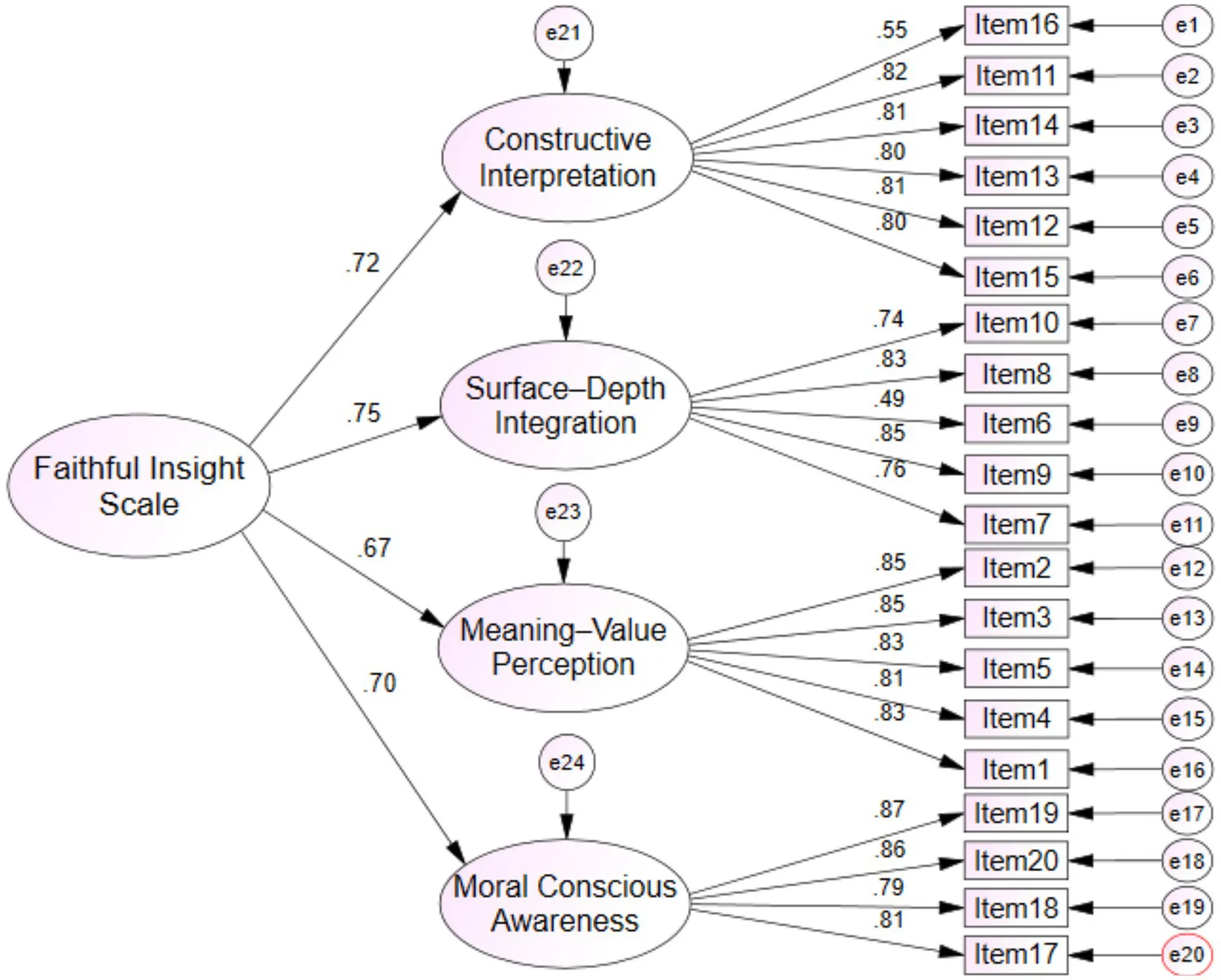
Path diagram of the final four-factor hierarchical CFA model.

All standardized item loadings and higher-order loadings exceeded the 0.50 salience threshold, confirming that the four-factor hierarchical structure identified in the EFA replicated in an independent confirmatory sample.

As recommended, the second-order (hierarchical) model was additionally compared against a correlated first-order model in which the four factors were permitted to freely intercorrelate without a higher-order factor. Because the second-order model is nested within the correlated first-order model with two fewer freely estimated parameters, this comparison indicates whether the higher-order factor adequately reproduces the pattern of inter-factor correlations. Fit indices for both models are presented in Table 7.

**Table 7:** Fit comparison of the second-order (hierarchical) and correlated first-order CFA models.

Fit index	Second-order (hierarchical) model	Correlated first-order model
*χ*^2^ (CMIN)	660.006	660.853
df	166	164
CMIN/df	3.975	4.030
GFI	0.925	0.925
AGFI	0.905	0.904
NFI	0.940	0.940
TLI	0.948	0.947
CFI	0.954	0.954
RMSEA [90% CI]	0.060 [0.055, 0.065]	0.060 [0.055, 0.065]
AIC	753.006	752.853
BIC	961.433	970.754

Fit indices for the two models converged closely (Δ*χ*^2^(2) = 0.85, ΔCFI = 0.000), with GFI, CFI, and RMSEA essentially identical and the second-order model showing marginally better CMIN/df, TLI, and BIC despite estimating two fewer parameters (44 vs. 46). Because the second-order model achieves equivalent fit with greater parsimony, and because it directly instantiates the hypothesized general factor (baṣīrah) explaining the correlations among the four dimensions, the second-order hierarchical model was retained as the preferred and theoretically consistent representation of the FIS factor structure.

### 3.4 Construct validity

Convergent and discriminant validity of the four-factor model were evaluated using composite reliability (CR), average variance extracted (AVE), maximum reliability (MaxR(H)), and the Fornell–Larcker criterion. As shown in Table 8, CR values ranged from 0.859 to 0.921, and AVE values ranged from 0.556 to 0.699, exceeding the recommended thresholds of 0.70 and 0.50, respectively, supporting adequate convergent validity for all four subscales. For the total scale, CR = 0.802 and AVE = 0.504, also exceeding recommended thresholds.

**Table 8:** CR, AVE, MaxR(H), and the Fornell–Larcker discriminant validity matrix.

Factor	CR	AVE	MaxR(H)	F1	F2	F3	F4
F1 (Constructive interpretation)	0.897	0.595	0.908	0.771	—	—	—
F2 (Surface–depth integration)	0.859	0.556	0.885	0.527	0.746	—	—
F3 (Meaning–value perception)	0.921	0.699	0.921	0.476	0.527	0.836	—
F4 (Moral conscious awareness)	0.900	0.693	0.905	0.527	0.508	0.452	0.833

Values in bold on the diagonal (0.771, 0.746, 0.836, 0.833) represent the square root of the AVE for each factor; off-diagonal values represent inter-factor correlations.

Discriminant validity was supported using the Fornell–Larcker criterion, whereby the square root of each factor’s AVE (presented on the diagonal of Table 8) exceeded its correlations with all other factors (presented in the off-diagonal cells). This pattern was observed for all four subscales, indicating that each factor captured a construct distinct from the others.

Taken together, the CR, AVE, and Fornell–Larcker results indicate that the four FIS subscales are internally coherent and empirically distinguishable from one another, supporting their interpretation as related but non-redundant facets of Faithful Insight.

### 3.5 Reliability

#### 3.5.1 Internal consistency

Internal consistency reliability for each subscale and for the total FIS score was estimated using Cronbach’s alpha (α), McDonald’s omega (ω), and Guttman’s lambda-2 (λ2). As presented in Table 9, all reliability coefficients exceeded 0.85 across the four subscales and the total scale, indicating excellent internal consistency. The mean inter-item correlation for the total scale was 0.387 (M = 2.925, 95% CI [2.873, 2.977]).

**Table 9:** Internal consistency reliability coefficients (α, ω, λ2) for the FIS subscales and total score.

Scale/subscale	α (95% CI)	ω (95% CI)	λ2 (95% CI)
F1–Constructive interpretation	0.895 [0.882, 0.909]	0.904 [0.894, 0.914]	0.903 [0.889, 0.916]
F2–Surface–depth integration	0.855 [0.838, 0.872]	0.873 [0.859, 0.887]	0.869 [0.852, 0.885]
F3–Meaning–value perception	0.920 [0.909, 0.932]	0.920 [0.912, 0.929]	0.920 [0.909, 0.932]
F4–Moral conscious awareness	0.899 [0.884, 0.914]	0.899 [0.888, 0.911]	0.899 [0.885, 0.914]
Total FIS score	0.927 [0.917, 0.936]	0.928 [0.921, 0.935]	0.930 [0.922, 0.939]

α, Cronbach’s alpha; ω, McDonald’s omega; λ2, Guttman’s lambda-2. Values in brackets represent 95% confidence intervals.

All internal consistency estimates exceeded the conventional 0.80 benchmark, indicating minimal measurement error attributable to item heterogeneity within each subscale.

#### 3.5.2 Test–retest reliability

Temporal stability of the FIS was examined in a subsample of 140 participants who completed the scale twice, with a 16-day interval between administrations. Following recommended practice for temporal stability estimation, the Intraclass Correlation Coefficient (ICC) was computed for each subscale and for the Total FIS score using a two-way mixed-effects model with absolute agreement, rather than Pearson correlation, as ICC additionally accounts for systematic shifts in mean level between administrations. Single-measures and average-measures ICCs, with 95% confidence intervals, are presented in Table 10. The Total FIS score demonstrated excellent temporal stability (single-measures ICC = 0.873, 95% CI [0.794, 0.918]; average-measures ICC = 0.932, 95% CI [0.885, 0.957]; *F*(139,139) = 17.050, *p* < 0.001). Subscale-level ICCs ranged from good (Constructive Interpretation, ICC = 0.620) to excellent (Surface–Depth Integration, ICC = 0.884), with all coefficients statistically significant (*p* < 0.001), indicating acceptable to excellent temporal stability across the FIS dimensions and supporting the reliability of the Total FIS score in particular.

**Table 10:** Test–retest reliability: intraclass correlation coefficients between Time 1 and Time 2 scores (*N* = 140, 16-day interval).

FIS dimension	ICC (single measures)	95% CI	ICC (average measures)	95% CI	*F*(139, 139)
Constructive interpretation	0.620	[0.504, 0.714]	0.766	[0.670, 0.833]	4.404***
Surface–depth integration	0.884	[0.832, 0.919]	0.938	[0.908, 0.958]	17.540***
Meaning–value perception	0.730	[0.612, 0.811]	0.844	[0.760, 0.896]	7.089***
Moral conscious awareness	0.872	[0.826, 0.907]	0.932	[0.904, 0.951]	14.525***
Total FIS	0.873	[0.794, 0.918]	0.932	[0.885, 0.957]	17.050***

***p < 0.001. ICCs estimated from a two-way mixed-effects model, absolute agreement definition. Single-measures ICC reflects the reliability of a single administration; average-measures ICC reflects the reliability of the mean of the two administrations. Interpreted per conventional benchmarks: < 0.50 poor, 0.50–0.75 moderate, 0.75–0.90 good, > 0.90 excellent (Koo and Li, 2016).

Constructive Interpretation showed comparatively lower (though still good) single-measures stability than the other three subscales, suggesting this dimension may be somewhat more sensitive to momentary contextual factors, whereas the Total FIS score and the remaining subscales showed good-to-excellent stability; this pattern is revisited in the Limitations.

## 4 Discussion

The present study successfully developed and psychometrically validated the FIS among Al-Azhar University students, yielding a robust four-factor hierarchical structure encompassing Constructive Interpretation, Surface–Depth Integration, Meaning–Value Perception, and Moral Conscious Awareness. The four-factor hierarchical CFA model demonstrated acceptable fit indices (*χ*^2^/df = 3.98, RMSEA = 0.060, CFI = 0.954), substantially outperforming the unidimensional alternative. Convergent validity was supported by CR values ranging from 0.859 to 0.921 and AVE values exceeding 0.50 across all subscales. Internal consistency was excellent, with Cronbach’s α, McDonald’s ω, and Guttman’s λ₂ all surpassing 0.85. Test–retest reliability for the total FIS score was strong (r = 0.833), collectively indicating that the FIS constitutes a psychometrically sound instrument for measuring faith-based insight in Muslim university populations.

The four factor names were chosen to directly reflect the facet of the conceptual model (Figure 1) theorized to underlie each dimension. Constructive Interpretation corresponds to tafakkur-based reframing of adversity through ʿaql; Surface–Depth Integration corresponds to the qalb’s theorized capacity to discern meaning beneath apparent circumstance; Meaning–Value Perception corresponds to muḥāsabah applied to evaluating the significance of experience; and Moral Conscious Awareness corresponds to murāqabah as ongoing self-monitoring against revealed moral standards. That all four first-order factors loaded substantially (0.673–0.745) onto a single higher-order factor empirically supports the theoretical premise that these facets jointly constitute baṣīrah, providing correspondence between the *a priori* conceptual model and the obtained factor structure.

The FIS addresses a critical methodological gap consistently identified in prior scholarship. Existing instruments measuring insight, religiosity, and spirituality among Muslim populations have been predominantly rooted in Western secular epistemological frameworks ill-suited to capturing faith-integrated cognition (Dasti and Sitwat, 2014; Haque et al., 2016). Direct adaptations of Western tools have demonstrated limited construct validity with Muslim respondents, as Islamic psychology conceptualizes cognition and self-awareness through distinctly Qur’anic dimensions that secular frameworks systematically overlook (Mitha, 2020; Tanhan and Young, 2021). While prior Arabic psychometric efforts have advanced measurement of resilience, emotional intelligence, and clinical insight (Fekih-Romdhane et al., 2024a, 2024b; Mohamed et al., 2026), none addressed faith-grounded cognitive insight. The FIS thus complements existing Islamic psychological instruments (Bukhori et al., 2022; Ghorbani et al., 2014; Nazam et al., 2022) by offering an indigenously grounded, empirically validated measure within Arab-Islamic quantitative research.

The FIS carries meaningful theoretical and applied implications. Theoretically, it operationalizes classical Islamic constructs — nafs, qalb, ʿaql, and rūḥ — within a contemporary psychometric framework, advancing the integration of Islamic epistemology and empirical psychological science. Practically, counselors, educators, and mental health practitioners working within Muslim contexts gain a validated tool for assessing faith-based insight as a psychological resource. Within Al-Azhar and analogous Islamic educational institutions, the FIS may inform pastoral and academic support programs by identifying students with lower faith-integrated reflective capacity who may benefit from targeted spiritual and psychological interventions. The scale’s hierarchical structure also permits both subscale-level profiling and total-score utility, enhancing its flexibility for diverse research and clinical applications.

Several limitations warrant acknowledgment. The sample was exclusively drawn from Al-Azhar University students, limiting generalizability to broader Muslim populations with varying levels of religious immersion and educational backgrounds. The cross-sectional design precludes causal inference regarding the developmental trajectory of Faithful Insight across the lifespan. Social desirability bias may have influenced self-reported responses within a religiously saturated institutional environment. Additionally, the test–retest interval of 16 days, while appropriate for stability estimation, does not capture longer-term temporal consistency. The absence of convergent validity evidence against established external criterion measures further constrains conclusions regarding nomological validity. Although gender, residence, academic major, and faculty were available as grouping variables, measurement invariance testing (or, alternatively, a MIMIC model) across these groups was not conducted in the present study; consequently, it remains unknown whether FIS scores can be validly compared across these subgroups, and this is recommended as a priority for future psychometric work on the scale. These limitations should be carefully considered when interpreting the present findings and generalizing the FIS beyond the validated population.

Future research should prioritize validating the FIS across diverse Muslim populations, including non-university adults, clinical samples, and Muslim communities in Western and non-Arab national contexts, to evaluate cross-cultural measurement invariance. Longitudinal designs would elucidate developmental changes in Faithful Insight across academic and life transitions. Establishing convergent validity through correlations with theoretically related constructs — including religiosity, psychological well-being, self-regulation, and moral reasoning — would strengthen the nomological network of the FIS. Criterion-related validity studies examining associations between FIS scores and behavioral outcomes such as academic resilience, prosocial behavior, and mental health would further demonstrate applied utility. Rasch modeling could complement classical test theory analyses, providing item-level diagnostic information to further refine the instrument.

## 5 Conclusion

The study introduces the FIS as a theoretically grounded and psychometrically robust instrument for measuring faith-based cognitive and spiritual insight among Muslim university students. The FIS demonstrated strong structural validity through a well-fitting four-factor hierarchical model, supported by evidence of convergent and discriminant validity, excellent internal consistency, and adequate temporal stability. By authentically integrating Islamic epistemological principles with rigorous psychometric methodology, the FIS addresses a substantive gap in Arab-Islamic psychological measurement. It offers researchers and practitioners a culturally congruent tool with significant potential for advancing theory and evidence-based practice in Islamic psychology, Muslim mental health, and faith-integrated educational settings across diverse international contexts.

## Data Availability

The raw data supporting the conclusions of this article will be made available by the authors, without undue reservation.
